# Nucleotide excision repair genes shaping embryonic development

**DOI:** 10.1098/rsob.190166

**Published:** 2019-10-30

**Authors:** Sofia J. Araújo, Isao Kuraoka

**Affiliations:** 1 Department of Genetics, Microbiology and Statistics, Faculty of Biology, University of Barcelona, 08028 Barcelona, Spain; 2 Institute of Biomedicine, University of Barcelona (IBUB), Barcelona, Spain; 3 Department of Chemistry, Faculty of Science, Fukuoka University, 8-19-1 Nanakuma, Jonan-ku, Fukuoka 814-0180, Japan

**Keywords:** nucleotide excision repair, development, embryo, central nervous system, xeroderma pigmentosum, Cockayne syndrome

## Abstract

Nucleotide excision repair (NER) is a highly conserved mechanism to remove helix-distorting DNA lesions. A major substrate for NER is DNA damage caused by environmental genotoxins, most notably ultraviolet radiation. Xeroderma pigmentosum, Cockayne syndrome and trichothiodystrophy are three human disorders caused by inherited defects in NER. The symptoms and severity of these diseases vary dramatically, ranging from profound developmental delay to cancer predisposition and accelerated ageing. All three syndromes include developmental abnormalities, indicating an important role for optimal transcription and for NER in protecting against spontaneous DNA damage during embryonic development. Here, we review the current knowledge on genes that function in NER that also affect embryonic development, in particular the development of a fully functional nervous system.

## 1. Human syndromes and NER deficiencies

The genome of all living beings exists in a dynamic equilibrium between ongoing DNA damage and reversal of the damage by DNA repair pathways. Multiple DNA repair mechanisms have evolved to shelter organisms from the continuous genotoxic stress induced by both intrinsic and extrinsic agents [1]. These agents can vary from cellular metabolites, such as reactive oxygen species (ROS), to environmental contaminants and ultraviolet (UV) radiation from the Sun [2]. DNA repair pathways can repair almost all possible DNA lesions created by these damaging agents. Consequently, a decrease in the cell's DNA repair capacity ultimately manifests itself in the form of mutagenesis, carcinogenesis, cellular senescence or cell death, and is implicated in a number of human diseases [3].

The disclosure of the intricacies of DNA repair has been made possible by the early description of human familial disease syndromes and by the more recent investigation of their genetic and molecular bases. The role of large protein complexes and the significance of their cellular localization are common features of many of the biochemical mechanisms involved. One of these DNA repair mechanisms is nucleotide excision repair (NER), which is responsible for removing a large variety of DNA lesions, including those helix-destabilizing DNA lesions induced by UV radiation [4]. There are two subclasses of NER. One is the global genome nucleotide excision repair (GG-NER), which removes lesions throughout the genome regardless of whether any specific sequence is transcribed or not. The other is the transcription-coupled nucleotide excision repair (TC-NER), which refers to the faster removal of damage from the transcribed strands of active genes.

Eukaryotic NER is a highly conserved multi-step process involving many different proteins whose molecular mechanism of action has been described in detail [5–9]. Alterations in NER genes are associated with autosomal recessive human diseases, such as xeroderma pigmentosum (XP), Cockayne syndrome (CS) and trichothiodystrophy (TTD), whose symptoms involve skin cancer and developmental and neurological symptoms. Other human syndromes associated with mutations in proteins involved in NER are cerebro-oculo-facio-skeletal (COFS) syndrome, UV-sensitive syndrome (UVSS) and the rare combined XP/CS [10].

XP is a prototypical DNA repair disorder and is characterized by extreme sensitivity to UV light and a 2000-fold incidence in skin cancer. Patients who are severely affected by XP also experience late-onset neurological defects and some affected individuals have neurodevelopmental abnormalities [11]. In XP, the skin cancer-prone phenotype is readily explained by the inability of these patients to repair UV-induced DNA lesions in skin tissues exposed to sunlight. By contrast, patients with CS are not overly cancer prone, but they endure additional symptoms. CS is a multi-system disorder with pleiotropic effects and patients have severe neurological abnormalities (including myelination defects, calcification and microcephaly), mental retardation, growth and developmental abnormalities, lack of subcutaneous fat, hypogonadism, tooth decay, cataracts and shorter lifespans [12]. CS is also considered to be a premature ageing disorder with patients displaying progressive neurodegeneration [2]. TTD includes a spectrum of ectodermal abnormalities such as congenital ichthyosis, brittle hair and short stature. Some of the most affected patients have an increased incidence of skin cancers and a wide variety of central nervous system (CNS) abnormalities [13].

Seven complementation groups with defects in the NER pathway have been assigned genetically in XP (XP-A to XP-G). An eighth one, XP variant (XP-V), is proficient in NER, but carries mutations in the *POLH* gene, which encodes DNA polymerase *η* (eta), a translesion synthesis (TLS) polymerase that specializes in error-free replication of DNA containing UV lesions [14,15].

The defining CS factors are Cockayne syndrome A (CSA) and B (CSB) proteins, although the CS phenotype can also result from specific mutations in some XP genes (*XPB*, *XPD* and *XPG*). In addition, another related factor, named XPA-binding protein 2 (XAB2), has been isolated as an XPA-interacting protein in a yeast two-hybrid screen. XAB2, a protein containing tetracopeptide repeats (TRP), also interacts with CSA, CSB and RNA polymerase II (RNAP2) [16]. Specifically, in cells treated with DNA-damaging agents, there was an enhanced interaction of XAB2 with RNAP2 or XPA [17]. Human cells depleted of XAB2 by RNAi show defects in transcription elongation and pre-mRNA splicing as well as hypersensitivity to killing by UV light and decreased recovery of RNA synthesis after UV irradiation, indicating that XAB2 is a multi-functional factor involved in splicing, transcription and TC-NER [17].

The transcription factor TFIIH is a central component of both NER processes (GG-NER and TC-NER). Mutations of its subunits are associated with both XP and CS. Like XAB2, TFIIH acts in distinct cellular processes. First, it is an essential component of the basic RNAP2 transcription machinery. Second, it is a basic DNA-repair factor, which is required for all repair by the NER pathway. And third, it can stimulate the ligand-dependent phosphorylation and activation of some nuclear receptors [18,19]. Genes for two subunits of TFIIH, *XPB* and *XPD*, are mutated in some cases of XP and CS. XPG, another XP factor, is responsible for maintaining the integrity and function of TFIIH [18] and is involved in some forms of CS as well [20]. Hence, whereas XP is a disease more directly linked with the NER core reaction, CS is intrinsically connected with the transcriptional side of DNA repair and general transcription defects [21,22].

## 2. The NER reaction: global genome repair and transcription-coupled repair

Both GG-NER and TC-NER employ a common set of proteins but differ in their mode of DNA damage recognition. GG-NER requires detection of the damaged sites in DNA by the UV-damaged DNA-binding protein (UV-DDB) and a complex containing XP group C (XPC) protein, the human homologue of RAD23 (either of two paralogues RAD23A and RAD23B) and the centrosomal protein Centrin-2 (CETN2) [23–25]. As shown by cell-free systems and structural analysis, XPC interacts with damaged DNA and subsequently initiates the repair reaction [5,26,27]. Damage in the transcribed strand of active genes is repaired by TC-NER, which is initiated by a stalled RNAP2 during transcription and depends on recruitment of the ATP-dependent chromatin remodelling protein CS protein B (CSB) and the adaptor subunit for a CUL4A-based E3 ubiquitin ligase CS protein A (CSA) to the site of damage [19,28,29].

**Figure 1.**
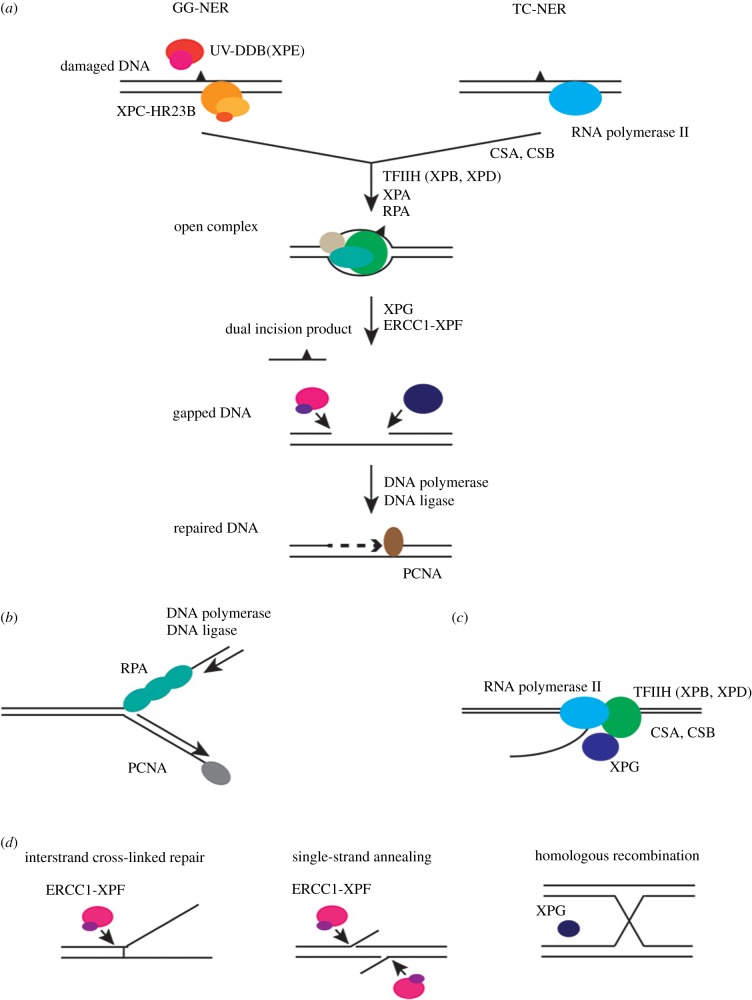
Schematic diagram of NER proteins involved in NER (TC-NER and GG-NER) and other pathways. (*a*) Schematic of the protein complexes involved in NER. Different recognition complexes operate during TC-NER and GG-NER. After the damage recognition step, the same protein complex is involved in damage excision and repair. NER factors also participate in replication (*b*), transcription (*c*) and other DNA repair pathways (*d*).

The NER reaction can be initiated by either of these two subpathways: GG-NER or TC-NER [30] (figure 1*a*). GG-NER can occur anywhere in the genome, whereas TC-NER is responsible for the accelerated repair of lesions in the transcribed strand of active genes. GG-NER is initiated by the GG-NER-specific factor XPC-RAD23B, in some cases with the help of UV-DDB [27]. TC-NER is initiated by RNAP2 stalled at a lesion with the help of TC-NER-specific factors CSA and CSB. Despite different beginnings, both pathways require the core NER factors to complete the excision process [10]. The core NER dual incision reaction has been reconstituted *in vitro* with purified factors using XPC-RAD23B, TFIIH, XPA, RPA, XPG and ERCC1-XPF [5]. Functional and structural studies revealed that XPC-RAD23B is the initial damage recognition factor in this system, as the presence of XPC-RAD23B is required for assembly of the other core NER factors and progression through the NER pathway both *in vitro* and *in vivo* [23,27,31,32].

The transcription and NER factor TFIIH is the next factor to join the NER complex and it is recruited by direct interaction with the XPC-RAD23B protein [2,33,34]. TFIIH consists of 10 subunits and can be divided up into the core (consisting of XPB, p52, p8, p62, p34, p44) and CAK (cyclin-activated kinase, consisting of CDK7, cyclin H and MAT1) complexes and the XPD protein that bridges the two [8]. The CAK complex dissociates from TFIIH and is not required for NER [5,35]. Of particular importance for the NER reaction are the two helicase subunits, XPB and XPD, which are known to open the DNA around the lesion [8,9,31]. The engagement of XPD with the lesion enables the full assembly of the pre-incision complex. XPA, RPA and XPG are next recruited to the site of the lesion independently of each other, and XPC-RAD23B departs from the complex at this point [36].

A central hub of the NER complex is XPA. It interacts with the TFIIH, RPA, XPC-RAD23B, DDB2, ERCC1-XPF and PCNA proteins, as well as with DNA. Through these interactions, XPA occupies a central role as an NER factor and probably works to make sure that all the NER factors are in the right place for the incision to occur (reviewed in [30]).

XPA interacts tightly with the ssDNA-binding protein RPA in the NER complex and the two are believed to cooperate in their association with DNA. There is evidence that RPA binds the non-damaged DNA strand, helping position the two endonucleases ERCC1- XPF and XPG on their substrate, the damaged DNA strand. The structure-specific endonuclease XPG is recruited through interaction with TFIIH. XPG, in fact, seems to be constitutively associated with TFIIH, at least for some of its roles in transcription [4,30,34]. Structural studies with recombinant human TFIIH show that XPB and XPD are stimulated by XPA and XPG and that these players change the mode of TFIIH from transcription to repair [9].

The complex consisting of TFIIH, XPA, RPA and XPG is relatively stable, and the dual excision reaction is only triggered once ERCC1-XPF joins the complex. ERCC1-XPF is recruited to NER complexes by interaction with the XPA protein. Once the two endonucleases are in place, dual incision at junctions between single-stranded and double-stranded DNA can be initiated [33,37]. Following the excision reaction, the lesion-containing oligonucleotide is released and the NER reaction finalizes with the resulting nucleotide single-stranded DNA gap being filled by DNA synthesis and ligation repair synthesis by DNA polymerases, associated factors and DNA ligase [5,30].

## 3. NER deficiencies and phenotype complexities

Many patients with mutations in NER or CS genes present developmental abnormalities at birth and may develop neurodegeneration later in life. Owing to the need for fast transcription during embryonic development [38,39] and in brain cells [40,41], many of these phenotypes may be due to the severely mutagenic and chromosome-destabilizing consequences of a stalled RNAP2. This could result in a transcriptional defect for critical genes, as well as a failure to accomplish TC-NER [42,43]. It has been hypothesized that TC-NER is more important for protecting non-dividing cells and neuronal function in the face of normal endogenous DNA damage [10,44]. This agrees with the general symptoms of XP-C patients, who have a defect in GG-NER but not in TC-NER and who present with neither developmental nor neurological abnormalities [45,46]. Interestingly, XP-A patients do not display obvious developmental phenotypes and do not seem to have widespread transcriptional impairment [47]. Affected individuals with mutations that completely ablate XPA function develop relatively normally, are born and may live for several decades. However, they often have various degrees of neurodegeneration [44]. Like other NER factors, XPA may have additional functions beyond NER. Recently, it was reported that XPA-deficient cells display mitochondrial dysfunction, with defects in mitophagy [46]. Mitochondrial dysfunction has been implicated in a number of pathophysiological processes such as ageing, neurodegenerative diseases, fertilization and embryonic development [48].

In fact, other NER factors are also involved not only in NER but also in replication, transcription and splicing (figure 1*b,c*). For instance, RPA was originally defined as a eukaryotic single-stranded DNA-binding protein essential for replication and an indispensable player in recombination (figure 1*b*). TFIIH is important for transcription initiation of RNAP2 during the expression of protein-coding genes and binds to a cyclin-activating kinase subcomplex for the cell cycle (figure 1*c*). Thus, the phenotypic complexity of patients with mutations in NER/CS genes might depend on a plethora of dysfunctional mechanisms (such as GG-NER, TC-NER, transcription, replication, recombination and splicing) fighting against DNA lesions in the context of the whole organism. In addition, we may speculate that some of the phenotype complexity could be due to neurodevelopment-specific DNA lesions recognized and repaired by NER. These still incompletely defined tissue-specific DNA lesions may have different effects on the organismal homeostasis.

In order to unravel the reason why NER-deficient patients develop neurodevelopmental abnormalities and neurodegeneration later in life, it is necessary to study possible embryonic-specific DNA lesions as well as which cellular mechanisms are impaired by them. A full understanding of the complex genotype/phenotype relationships of human DNA damage response disorders clearly requires further studies and suitable disease animal models [49,50].

## 4. NER and possible DNA lesions during embryonic development

As mentioned previously, human NER is the main pathway eliminating a wide variety of helix-destabilizing bulky DNA lesions that block DNA replication and transcription [1]. One important source of such DNA lesions is exposure to the UV component of sunlight, which generates photolesions (cyclobutane pyrimidine dimers (CPDs) and 6-4 pyrimidone photoproducts (6-4PPs)) in DNA. Cells from NER-deficient patients, that is, those with XP, CS or TTD, are extremely sensitive to UV light and patients with XP show an increased incidence of sunlight-induced skin cancers [2]. But what types of DNA damage may be responsible for the developmental abnormalities displayed by NER-deficient patients? UV radiation cannot generate photoinduced lesions in fetal or embryonic cells. So, sources of damage during development are most likely to be different, as NER eliminates not only UV-induced DNA lesions but also bulky DNA lesions such as the adducts induced by the anti-cancer drug cisplatin or mutagens like acetylaminofluorene [51]. Exposure to these carcinogenic substances may induce some of these NER-repairable lesions. However, these are neither very common nor a source of significant damage during human gestation.

Hence, DNA-damaging sources during embryonic development are most likely to be endogenous to cells, rather than exogenous. A spontaneous source of DNA damage inside patients' bodies is cellular generated ROS, such as superoxide and hydrogen peroxide, which produce hydroxyl radicals via the Fenton reaction that are highly reactive and cause various modified DNA bases [52]. Among them, 8-oxo-7,8-dihydroguanine (8-oxoG) is the most abundant and seems to play a major role in mutagenesis and in carcinogenesis. Interestingly, 8-oxoG is highly accumulated in the brain cells of patients with Alzheimer or Parkinson disease [53]. As a tissue, the brain is very sensitive to ROS, owing to its high oxygen consumption, about 20% of the whole body [54]. Thus, the brain is especially vulnerable to oxidative stress. In most cases, 8-oxoG is mainly removed from DNA by human base excision repair (BER) using 8-oxoguanine DNA glycosylase (OGG1), endonuclease III-like 1 (NTH1) and endonuclease VIII-like 1 (NEIL1) [55]. 8-oxoG is not a bulky, helix-destabilizing DNA lesion, but it has been reported that NER can also be involved in removing 8-oxoG from DNA [56].

Another important candidate for the endogenous generation of helix-distorting bulky DNA lesions by ROS is purine cyclodeoxynucleoside (cyPu) [52,57]. This type of lesion can block replication and it is unlikely to be removed by BER. Action of a glycosylase in BER would not be expected to release such cyPus, because the purine would remain attached by the 5′,8 carbon–carbon bond even after cleavage of the glycosyl bond. The cyPu lesions may be repaired by NER, which can remove oligonucleotides containing a DNA lesion by dual incision action. The lesions appear to be relatively abundant forms of DNA damage after exposure to ROS, introduced at 20–30% of the levels of the major lesions, although the relative rates of formation vary with experimental conditions. Thus, cyPu lesions in the brain might explain the progressive neurodegeneration seen in NER-deficient individuals [52,57]. Other candidate lesions for NER action are lipid peroxidation (LPO) product lesions and acetaldehyde-induced DNA lesions [58,59]. LPO products originate during normal cellular metabolism and generate protein and DNA adducts, which have detrimental effects in embryonic cells and can be repaired by NER [60,61]. Acetaldehyde is thought to cause a variety of DNA lesions and occurs naturally in various plants, ripe fruits and vegetables. In addition, drinking alcohol and smoking cigarettes can lead to high levels of acetaldehyde in the body that can be passed on to the developing fetus. Even without these environmental challenges, human cells are constantly exposed to acetaldehyde [58], and some acetaldehyde-induced DNA lesions might be repaired by NER. Interestingly, an acetaldehyde-GG cross-link resembles CPDs, 6-4PP and cisplatin-induced-GG adducts, and might be repaired as such. These lesions show an increase of GG-to-TT mutations in NER-deficient human XP cells [62]. Genome-wide analysis of sequence signatures indicates that GG-to-TT mutations are associated with cancer, suggesting that acetaldehyde in our body might induce DNA lesions [63]. During embryonic development, acetaldehyde can be detected in fetuses of alcoholic mothers and has been shown to have teratogenic effects [64,65].

By and large, it is unknown which kinds of DNA lesions cause developmental abnormalities in NER patients. Since NER, including both GG-NER and TC-NER, removes a wide variety of DNA lesions, it will be important to detect NER-repairable DNA lesions in cells during embryonic development.

## 5. NER and embryonic development

DNA repair is crucial both for dividing proliferating cells, in which lesions in DNA interfere with replication fork progression and may be converted into mutations upon replication, and for non-dividing differentiated cells, which sometimes have to maintain their genome integrity for the entire lifespan of the organism and have cell division-dependent checkpoints downregulated or switched off. In the first case, failure of DNA repair will induce mutations whereas in the second case it will give rise to an accumulation of DNA damage that can interfere with many cellular processes [45].

In actively proliferating cells, such as the cells of the early developing embryo, DNA repair is crucial for preventing the accumulation of mutations and synchronizing cell division [66,67]. Accordingly, it has been shown using the nematode *Caenorhabditis elegans* that early developmental stages are more sensitive to UV irradiation than later stages [68]. However, many developmental processes such as late organogenesis rely on fully differentiated cells, which are not actively dividing but frequently need to change their behaviours very rapidly, a process that relies on the fast transcription of many genes. Organ formation requires rapid cell proliferation, active gene transcription and a high rate of DNA metabolism, especially during the developmental stages. Thus, embryonic cells are likely to be sensitive to both global-genome and transcribed-strand damage with slower rates of transcription leading to embryonic lethality [69]. In addition, an increase in NER capacity accompanies cell differentiation, as shown by the upregulated transcription of genes encoding XPA, XPC, XPG and ERCC1-XPF during neuron and muscle cell differentiation [70]. Hence, the proteins involved in the two NER pathways, GG-NER and TC-NER, are probably necessary for proper embryonic development, from the oocyte to fully developed organismal stages. Embryonic development can progress to term in the complete absence of NER, as shown by the apparently normal development and size of XPA knockout mice and humans [71]. Of course, there are severe developmental abnormalities displayed by many patients with XP or CS [72]. These are likely to be a combined effect of compromised transcription and DNA repair. In addition, if we consider that increased risk of developing cancer is based on intrinsic developmental defects at the molecular and cellular level, then most known DNA repair deficiencies are associated with significant developmental abnormalities.

As for many other biological studies, the ability to understand the interplay between NER and developmental processes requires appropriate model organisms. So far, much has been learned about human embryonic development and physiology through the study of model animals, which have particular advantages for laboratory research. There are many reasons for using them. Research on humans and other primates is expensive and limited by ethical considerations whereas the most commonly studied model animals are relatively inexpensive to maintain and are well suited for experimental manipulation [73]. In addition, recent research has shown that there is a remarkable degree of similarity in the developmental mechanisms of all animals. In developing model organism embryos, not only individual genes and proteins but also entire signalling pathways and cell behaviours appear highly conserved. This means that, although the embryology of simpler animals might appear superficially very different from that of humans, knowledge gained from those models can often be applied directly to understanding human developmental mechanisms. Furthermore, many of the known human disease-causing mutations are hypomorphic and animal models are the ideal way to study the effects of amorphic mutations during development, since many of these null mutants result in embryonic lethality. Therefore, research on the involvement of NER proteins in developmental biology has been largely done using model organisms.

## 6. Embryonic development without NER factors: survival and phenotypes

**Figure 2.**
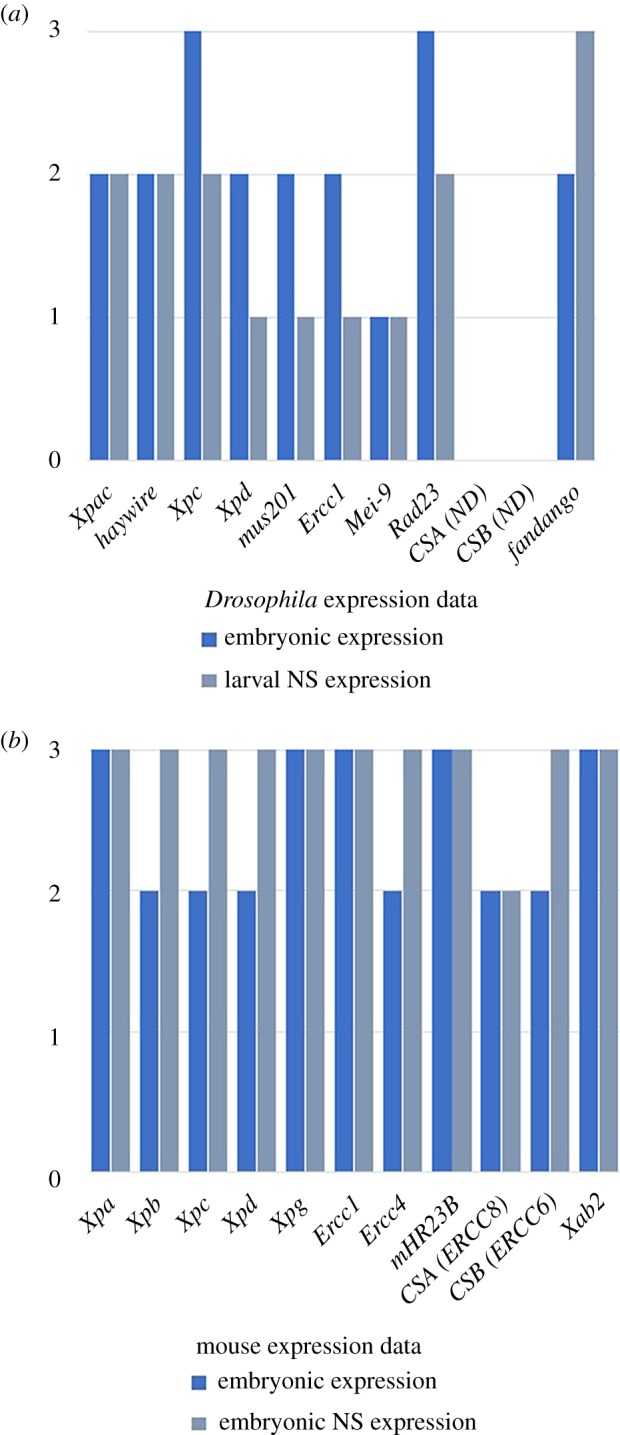
Embryonic and nervous system (NS) expression of NER genes in *Drosophila melanogaster* and *Mus musculus.* Graphical representation of transcript expression during embryonic development, according to high-throughput expression data. (*a*) *D. melanogaster* expression data from ModENCODE (www.modencode.org) tissue and temporal expression data; (*b*) *M. musculus* expression data from Expression Atlas (www.ebi.ac.uk/gxa/home) embryonic and tissue expression data. Data mining was performed according to all developmental stages (embryonic expression) and specific nervous system expression (larval expression for *Drosophila* and embryonic expression for mouse). Arbitrary values were attributed according to expression levels (1, low; 2, moderate; and 3, high) and plotted in parallel.

It has long been known that many NER proteins are actively expressed in many tissues during embryonic development even in the absence of external DNA-damaging agents [74–76]. NER genes, together with other DNA repair pathway genes, are expressed from early stages of embryonic development [77]. In widely studied model organisms such as *Mus musculus* or *Drosophila melanogaster*, expression of NER factors has been observed during development, ubiquitously in the whole organism or in specific tissues (table 1 and figure 2).

**Table 1.** Known embryonic transcript expression of NER transcripts in *Mus musculus* according to the Gene eXpression Database (GXD; http://www.informatics.jax.org/expression.shtml) and *Drosophila melanogaster* according to the Berkeley Drosophila Genome Project (BDGP; https://insitu.fruitfly.org). ND, not identified in this species; GUDMAP, GenitoUrinary Development Molecular Anatomy Project.

mouse gene	embryonic expression	reference	*Drosophila* gene	embryonic expression	reference
*Xpa*	limb bud	[78]	*Xpac*	ventral nerve cord	[79]
*Xpb*	ubiquitous; nervous system and liver	[74]	*haywire*	ubiquitous	[80] and BDGP
*Xpc*	no expression data	—	*Xpc*	faint ubiquitous	[80] and BDGP
*Xpd*	nervous system, eye and liver	[81]	*Xpd*	ubiquitous (nuclear)	[82]
*Xpg*	nervous system	[83]	*mus201*	no available data	—
*Ercc1*	nervous system	[84]	*Ercc1*	no available data	—
*Ercc4*	nervous system	[83]	*Mei-9*	no available data	—
*mHR23B*	nervous system; genitourinary system	[81] and GUDMAP	*Rad23*	faint ubiquitous	[85]
*CSA (Ercc8)*	cranium	[86]	CSA (ND)	—	—
*CSB (Ercc6)*	genitourinary system	GUDMAP	CSB (ND)	—	—
*Xab2*	nervous system	[81]	*fandango*	ubiquitous	[38]

One of the earliest observations of a strong influence of NER factors in embryonic development was a report showing that null mice lacking ERCC1 died before weaning [87]. Since then, other null mutations in NER genes have proven to be embryonic lethal in different species, suggesting a strong need for some of these factors during development [71] (table 2). When not lethal, many of these null mutations, such as *XPA* and *CSB*, induce growth retardation [75,105], another hint to their important functions during development (table 2). When some of these mutations are combined in the same animal, they give rise to stronger phenotypes, suggesting genetic interactions during developmental processes between many of these factors [71]. For instance, mice lacking both XPA and CSB displayed severe growth retardation, ataxia and motor dysfunction during early postnatal development, suggesting that these genes may have additive roles during nervous system development [106].

**Table 2.** Requirements of NER genes during development inferred by the analysis of null mutations in *Mus musculus* and *Drosophila melanogaster*.

mouse gene	phenotype	*Drosophila* gene	phenotype	reference
*Xpa*	viable; develop normally	*Xpac*	no mutant developmental data	[88]
*Xpb*	embryonic lethal	*haywire*	embryonic lethal; CNS defects	[89,90]
*Xpc*	viable; develop normally	*Xpc*	no mutant developmental data	[91,92]
*Xpd*	pre-implantation lethality	*Xpd*	embryonic lethal; early mitotic division defects	[93,94]
*Xpg*	mice are viable but die before weaning	*mus201*	no mutant developmental data	[95,96]
*Ercc1*	viable but growth failure and death before weaning	*Ercc1*	no mutant developmental data	[87,97]
*Ercc4*	severe postnatal growth defect with death approximately three weeks after birth	*Mei-9*	no mutant developmental data	[98,99]
*mHR23B*	impaired embryonic development; prenatal and early postnatal death (90%)	*Rad23*	no mutant developmental data	[100]
*CSA (Ercc8)*	viable; minor postnatal growth retardation and neurological defects	not identified	—	[101]
*CSB (Ercc6)*	viable; minor postnatal growth retardation and neurological defects	not identified	—	[102]
*Xab2*	embryonic lethal	*fandango*	embryonic lethal; organogenesis defects	[103,104]

One of the crucial factors in NER is TFIIH, which is also one of the factors that bridges the two human syndromes XP and CS. Of the many TFIIH subunits, only XPB and XPD can be involved in both XP and CS. In *Drosophila*, loss of *haywire (hay)*, the gene homologous to XPB, leads to male sterility, CNS defects and UV sensitivity, not unlike human XPB/CS patients [89] (table 2). Hay is expressed in several stages of development and *hay* mutant embryos display phenotypes that range from completely disordered ventral nerve cords (VNCs) to VNCs with only a few broken commissures [89]. Transgenic flies carrying human-like alleles with mutations reported in human patients reproduce these defects, suggesting that *Drosophila* is a good model for these studies [107]. Another existing model for another TFIIH subunit, XPD, has been reported in *Drosophila*, allowing for different human mutations to be tested during development [108]. This *Drosophila* model revealed an Xpd function in cell cycle coordination which is affected by XP/CS and TTD mutations [108]. The two XP/CS alleles G47R and G675R, as well as the TTD allele R722 W, showed the highest frequency of asynchronous waves of all the *xpd* mutants in this *Drosophila* model. Human patients with these mutations display severe neurological abnormalities, reduced growth, and delayed and defective development, correlating the degree of neurological abnormalities with asynchronous waves of cell division [108]. XPB and XPD mutants have also been analysed in other model organisms such as zebrafish or mouse (table 1). Overall, these two TFIIH subunits have been shown to be important for embryonic development across species [90,109]. XPB and XPD being subunits of TFIIH implies that their involvement in embryonic development is also due to their direct effects in transcription. Crippled transcription of key developmental genes might be responsible for the observed developmental phenotypes [110].

One more factor shown to be involved in both XP and CS is the endonuclease XPG [111]. Mice carrying truncated forms of XPG, generally associated with CS, exhibited postnatal growth failure and premature death, similar to the clinical hallmarks of CS despite apparent normal development [95]. In *Drosophila*, mutant flies are defective in NER and hypersensitive to UV radiation as the homozygous mutant mice. However, in contrast to these, the two *Drosophila* mutants are viable and fertile in the absence of exogenous DNA-damaging agents [112,113]. XPG has also been found to be a partner of BRCA1 and BRCA2 in maintaining genomic stability through homologous recombination (HRR) [114] (figure 1*d*). The role of this endonuclease in HRR suggests that this player has important roles in genome stability and may explain some of the phenotypes and clinical consequences associated with its loss of function.

The other NER endonuclease is ERCC1, which when mutated in mice leads to attenuated growth, resulting in cachectic dwarfism during the second week of life and premature death before postnatal day 35 [97]. This severe growth retardation was shown to originate from defective transcription initiation of developmental gene expression programmes [97]. In addition, ERCC1 has also been implicated in double-strand break, interstrand cross-link (ICL) and base excision repair [37] (figure 1*d*). This suggests that, as in the case of XPG, the developmental defects associated with mutations in ERCC1 may be due to transcriptional impairment as a consequence of faulty chromatin remodelling or other defective DNA damage responses, rather than to a direct effect of NER in the developmental programme. Interestingly, a metabolic connection was found between defects in ERCC1 and patients' phenotypes, suggesting an association between ERCC1 and organismal homeostasis and energy balance [115].

CSA (ERCC8) and CSB (ERCC6) are two factors directly associated with CS and mice deficient for either of these genetically mimic CS in humans [116]. However, when analysed at birth these mutants do not seem to show any developmental abnormalities, leading to the conclusion that CSA and CSB are not directly involved in any developmental process [116]. To gain further insight into these mutants and their effects in whole-organism homeostasis, various double mutant combinations were generated between CS and XP factors (reviewed in [116]). Of these, it is interesting to pinpoint the Csb/Xpa and Csb/Xpc double mutant mice, which had a very short lifespan and severe pathology in multiple tissues. In some litters, there was perinatal death and in others defects started very early in postnatal life. In addition, double mutant pups showed progressive development of ataxia and other motor dysfunctions, which correlated with smaller cerebella with a reduced number of granule cells [106]. In addition, *Csb−/−* embryonic and adult neural precursors exhibited defective self-renewal, and neurons differentiated *in vitro* from *Csb−/−* neural precursors, which had been irradiated with UV, exhibited defective neurite outgrowth [117]. Taken together, these data point at an active role of CSB during neurogenesis and the morphogenesis of the nervous system.

Irregularities in the regulation of transcription might account for many of the somatic features associated with CS, including neurological symptoms. CSB may have an important role in the transcriptional programmes that govern the plasticity and the maintenance of the CNS during early life [118]. Neurogenesis occurs both during embryonic development and later in life and failure to accomplish this process may lead to neurodevelopmental and neurodegeneration phenotypes. Accordingly, CSB deficiency has been shown to affect neuronal differentiation, suggesting that patients with CS are less able to support brain plasticity and repair events [119].

CS complementation genes *CSA* and *CSB* have also been studied in non-vertebrate models such as *C. elegans*. Mutations in the nematode *csa-1* and *csb-1* genes lead to developmental growth defects and UV sensitivity and both genes are expressed throughout embryonic development [120–122]. In *Drosophila*, neither *CSA* nor *CSB* homologues are present, despite their presence in many insect species [123]. It was reported in the past that repair of the transcribed strand occurs at the same speed as that of the non-transcribed strand both in embryonically derived cells and in brain tissue [124,125]. Lack of clear gene homology and biochemical data on GG versus TC-NER has led to the conclusion that *Drosophila* does not carry out TC-NER [126]. However, this is still under discussion, as flies would be the only model organism not to be able to actively repair highly transcribed genes. An alternative explanation is that there is CSB-independent TC-NER in *Drosophila* as has been shown in yeast [127,128]. Furthermore, the lack of differences between actively transcribed and non-transcribed genes in *Drosophila* was experimentally done using the *white* (*w*) gene as a control non-transcribed gene in both embryos and larval brains [124,125]. However, *w* expression could be detected in the same brain tissues where the comparison between repair of different strands was made [125]. And later reports have shown that, indeed, *w* is expressed both in embryos and in larvae and has pleiotropic effects in the whole organism [129,130]. Moreover, XAB2, a binding partner for CSA and CSB [17], has been recently identified in *Drosophila*, where it was named *fandango* (*fand*), and has been shown to be involved in embryonic pre-mRNA splicing and organogenesis [38,103]. As in *Drosophila*, null mutants for XAB2 in mice are embryonic lethal, pointing at the important function of this gene during development [104]. So, the quest for factors controlling possible TC-NER in *Drosophila* is still on.

Overall, all current data seem to point out that many factors involved in NER are also important during embryonic development. However, during these studies, analysis of developmental defects was done without the challenge of exogenous DNA repair, during normal development, taking into account only endogenous levels of DNA damage. Hence, the effects of NER/CS mutations in development are mostly analysed under conditions that mimic low levels of DNA damage. Stronger phenotypes are attained if embryos are subjected to exogenous DNA damage. A study using *C. elegans* has revealed that DNA ICLs lead to developmental arrest and tissue defects in mutants for NER proteins [131], revealing the importance of NER in embryos subjected to extra sources of DNA damage.

Taken all together, the role of NER in embryonic development is not yet well understood; however, a number of clues have surfaced indicating that efficient repair of endogenous damage may be crucial to normal development. It seems that factors involved in GG-NER as well as TC-NER are required for proper development. The close association between RNA transcription and the repair of bulky lesions on the transcribed strand of the DNA suggests that efficient repair of lesions that block transcription is crucial for sustaining the complex cellular balance required for proper development. Hence, it is likely that this aspect of NER is essential for proper development.

We have come a long way, but much more information is needed to determine to what extent NER of endogenous or environmentally induced DNA damage is influential during the correct formation of an organism and what are the cross-talks between the NER machinery and the developmental programmes.

## Supplementary Material

Reviewer comments

## Data accessibility

This article has no additional data.

## References

[1] Lindahl T, Wood RD 1999 Quality control by DNA repair. Science 286, 1897–1905. (10.1126/science.286.5446.1897) 10583946

[2] Friedberg EC, Walker GC, Siede W, Wood RD, Schultz RA, Ellenberger T 2006 DNA repair and mutagenesis, 2nd edn Washington, DC: ASM Press.

[3] O'Driscoll M 2012 Diseases associated with defective responses to DNA damage. Cold Spring Harbor Perspect. Biol. 4, a012773 (10.1101/cshperspect.a012773) PMC3504433 23209155

[4] Spivak G 2015 Nucleotide excision repair in humans. DNA Repair 36, 13–18. (10.1016/j.dnarep.2015.09.003) 26388429 PMC4688078

[5] Araújo SJ, Tirode F, Coin F, Pospiech H, Syväoja JE, Stucki M, Hübscher U, Egly JM, Wood RD 2000 Nucleotide excision repair of DNA with recombinant human proteins: definition of the minimal set of factors, active forms of TFIIH, and modulation by CAK. Genes Dev. 14, 349–359. 10673506 PMC316364

[6] Araujo SJ, Wood RD 1999 Protein complexes in nucleotide excision repair. Mutat. Res. 435, 23–33. (10.1016/S0921-8777(99)00042-7) 10526214

[7] Feltes BC, Bonatto D 2015 Overview of xeroderma pigmentosum proteins architecture, mutations and post-translational modifications. Mutat. Res. Rev. Mutat. Res. 763, 306–320. (10.1016/j.mrrev.2014.12.002) 25795128

[8] Greber BJ, Toso DB, Fang J, Nogales E 2019 The complete structure of the human TFIIH core complex. eLife 8, e44771 (10.7554/eLife.44771) 30860024 PMC6422496

[9] Kokic G, Chernev A, Tegunov D, Dienemann C, Urlaub H, Cramer P 2019 Structural basis of TFIIH activation for nucleotide excision repair. Nat. Commun. 10, 2885 (10.1038/s41467-019-10745-5) 31253769 PMC6599211

[10] Liakos A, Lavigne MD, Fousteri M 2017 Nucleotide excision repair: from neurodegeneration to cancer. Adv. Exp. Med. Biol. 1007, 17–39. (10.1007/978-3-319-60733-7_2) 28840550

[11] Lehmann J, Seebode C, Martens MC, Emmert S 2018 Xeroderma pigmentosum - facts and perspectives. Anticancer Res. 38, 1159–1164. 29374753 10.21873/anticanres.12335

[12] Cleaver JE, Lam ET, Revet I 2009 Disorders of nucleotide excision repair: the genetic and molecular basis of heterogeneity. Nat. Rev. Genet. 10, 756–768. (10.1038/nrg2663) 19809470

[13] Faghri S, Tamura D, Kraemer KH, Digiovanna JJ 2008 Trichothiodystrophy: a systematic review of 112 published cases characterises a wide spectrum of clinical manifestations. J. Med. Genet. 45, 609–621. (10.1136/jmg.2008.058743) 18603627 PMC3459585

[14] Masutani C et al. 1999 The XPV (xeroderma pigmentosum variant) gene encodes human DNA polymerase η. Nature 399, 700–704. (10.1038/21447) 10385124

[15] Lehmann AR, McGibbon D, Stefanini M 2011 Xeroderma pigmentosum. Orphanet J. Rare Dis. 6, 70 (10.1186/1750-1172-6-70) 22044607 PMC3221642

[16] Nakatsu Y et al. 2000 XAB2, a novel tetratricopeptide repeat protein involved in transcription-coupled DNA repair and transcription. J. Biol. Chem. 275, 34 931–34 937. (10.1074/jbc.M004936200) 10944529

[17] Kuraoka I et al. 2008 Isolation of XAB2 complex involved in pre-mRNA splicing, transcription, and transcription-coupled repair. J. Biol. Chem. 283, 940–950. (10.1074/jbc.M706647200) 17981804

[18] Ito S, Kuraoka I, Chymkowitch P, Compe E, Takedachi A, Ishigami C, Coin F, Egly JM, Tanaka K 2007 XPG stabilizes TFIIH, allowing transactivation of nuclear receptors: implications for Cockayne syndrome in XP-G/CS patients. Mol. Cell 26, 231–243. (10.1016/j.molcel.2007.03.013) 17466625

[19] Svejstrup JQ 2002 Mechanisms of transcription-coupled DNA repair. Nat. Rev. Mol. Cell Biol. 3, 21–29. (10.1038/nrm703) 11823795

[20] Shiomi N, Kito S, Oyama M, Matsunaga T, Harada YN, Ikawa M, Okabe M, Shiomi T 2004 Identification of the XPG region that causes the onset of Cockayne syndrome by using *Xpg* mutant mice generated by the cDNA-mediated knock-in method. Mol. Cell. Biol. 24, 3712–3719. (10.1128/MCB.24.9.3712-3719.2004) 15082767 PMC387744

[21] Newman JC, Bailey AD, Weiner AM 2006 Cockayne syndrome group B protein (CSB) plays a general role in chromatin maintenance and remodeling. Proc. Natl Acad. Sci. USA 103, 9613–9618. (10.1073/pnas.0510909103) 16772382 PMC1480455

[22] Lake RJ, Fan H-Y 2013 Structure, function and regulation of CSB: a multi-talented gymnast. Mech. Ageing Dev. 134, 202–211. (10.1016/j.mad.2013.02.004) 23422418 PMC3750219

[23] Batty D, Rapic'-Otrin V, Levine AS, Wood RD 2000 Stable binding of human XPC complex to irradiated DNA confers strong discrimination for damaged sites. J. Mol. Biol. 300, 275–290. (10.1006/jmbi.2000.3857) 10873465

[24] Dantas TJ, Wang Y, Lalor P, Dockery P, Morrison CG 2011 Defective nucleotide excision repair with normal centrosome structures and functions in the absence of all vertebrate centrins. J. Cell Biol. 193, 307–318. (10.1083/jcb.201012093) 21482720 PMC3080269

[25] Sugasawa K 2016 Molecular mechanisms of DNA damage recognition for mammalian nucleotide excision repair. DNA Repair (Amst). 44, 110–117. (10.1016/j.dnarep.2016.05.015) 27264556

[26] Sugasawa K, Ng JM, Masutani C, Iwai S, van der Spek PJ, Eker AP, Hanaoka F, Bootsma D, Hoeijmakers JHJ 1998 Xeroderma pigmentosum group C protein complex is the initiator of global genome nucleotide excision repair. Mol. Cell. 2, 223–232. (10.1016/S1097-2765(00)80132-X) 9734359

[27] Paul D, Mu H, Zhao H, Ouerfelli O, Jeffrey PD, Broyde S, Min J-H 2019 Structure and mechanism of pyrimidine-pyrimidone (6-4) photoproduct recognition by the Rad4/XPC nucleotide excision repair complex. Nucleic Acids Res. 47, 6015–6028. (10.1093/nar/gkz359) 31106376 PMC6614856

[28] Schärer OD 2008 Hot topics in DNA repair: the molecular basis for different disease states caused by mutations in TFIIH and XPG. DNA Repair (Amst). 7, 339–344. (10.1016/j.dnarep.2007.10.007) 18077223 PMC2246058

[29] Groisman R, Polanowska J, Kuraoka I, Sawada J-i, Saijo M, Drapkin R, Kisselev AF, Tanaka K, Nakatani Y 2003 The ubiquitin ligase activity in the DDB2 and CSA complexes is differentially regulated by the COP9 signalosome in response to DNA damage. Cell 113, 357–367. (10.1016/S0092-8674(03)00316-7) 12732143

[30] Schärer OD 2013 Nucleotide excision repair in eukaryotes. Cold Spring Harbor Perspect. Biol. 5, a012609 (10.1101/cshperspect.a012609) PMC3783044 24086042

[31] Evans E, Moggs JG, Hwang JR, Egly JM, Wood RD 1997 Mechanism of open complex and dual incision formation by human nucleotide excision repair factors. EMBO J. 16, 6559–6573. (10.1093/emboj/16.21.6559) 9351836 PMC1170260

[32] Volker M et al. 2001 Sequential assembly of the nucleotide excision repair factors *in vivo*. Mol. Cell. 8, 213–224. (10.1016/S1097-2765(01)00281-7) 11511374

[33] Evans E, Fellows J, Coffer A, Wood RD 1997 Open complex formation around a lesion during nucleotide excision repair provides a structure for cleavage by human XPG protein. EMBO J. 16, 625–638. (10.1093/emboj/16.3.625) 9034344 PMC1169665

[34] Araújo SJ, Nigg EA, Wood RD 2001 Strong functional interactions of TFIIH with XPC and XPG in human DNA nucleotide excision repair, without a preassembled repairosome. Mol. Cell. Biol. 21, 2281–2291. (10.1128/MCB.21.7.2281-2291.2001) 11259578 PMC86862

[35] Coin F, Oksenych V, Mocquet V, Groh S, Blattner C, Egly JM 2008 Nucleotide excision repair driven by the dissociation of CAK from TFIIH. Mol. Cell 31, 9–20. (10.1016/j.molcel.2008.04.024) 18614043

[36] Riedl T, Hanaoka F, Egly JM 2003 The comings and goings of nucleotide excision repair factors on damaged DNA. EMBO J. 22, 5293–5303. (10.1093/emboj/cdg489) 14517266 PMC204472

[37] Manandhar M, Boulware KS, Wood RD 2015 The ERCC1 and ERCC4 (XPF) genes and gene products. Gene 569, 153–161. (10.1016/j.gene.2015.06.026) 26074087 PMC4536074

[38] Guilgur LG, Prudêncio P, Sobral D, Liszekova D, Rosa A, Martinho RG 2014 Requirement for highly efficient pre-mRNA splicing during Drosophila early embryonic development. Elife 3, e02181 (10.7554/eLife.02181) 24755291 PMC3989599

[39] Martinho RG, Guilgur LG, Prudêncio P 2015 How gene expression in fast-proliferating cells keeps pace. Bioessays 37, 514–524. (10.1002/bies.201400195) 25823409

[40] Laposa RR, Cleaver JE 2001 DNA repair on the brain. Proc. Natl Acad. Sci. USA 98, 12 860–12 862. (10.1073/pnas.241519498) PMC60784 11698674

[41] Canugovi C, Misiak M, Ferrarelli LK, Croteau DL, Bohr VA 2013 The role of DNA repair in brain related disease pathology. DNA Repair (Amst) 12, 578–587. (10.1016/j.dnarep.2013.04.010) 23721970 PMC3720728

[42] Hanawalt PC, Spivak G 2008 Transcription-coupled DNA repair: two decades of progress and surprises. Nat. Rev. Mol. Cell Biol. 9, 958–970. (10.1038/nrm2549) 19023283

[43] Gregersen LH, Svejstrup JQ 2018 The cellular response to transcription-blocking DNA damage. Trends Biochem. Sci. 43, 327–341. (10.1016/j.tibs.2018.02.010) 29699641 PMC5929563

[44] Kraemer KH, Patronas NJ, Schiffmann R, Brooks BP, Tamura D, DiGiovanna JJ 2007 Xeroderma pigmentosum, trichothiodystrophy and Cockayne syndrome: a complex genotype-phenotype relationship. Neuroscience 145, 1388–1396. (10.1016/j.neuroscience.2006.12.020) 17276014 PMC2288663

[45] Iyama T, Wilson DM 2013 DNA repair mechanisms in dividing and non-dividing cells. DNA Repair (Amst). 12, 620–636. (10.1016/j.dnarep.2013.04.015) 23684800 PMC3720834

[46] Fang EF, Scheibye-Knudsen M, Brace LE, Kassahun H, Sengupta T, Nilsen H, Mitchell JR, Croteau DL, Bohr VA 2014 Defective mitophagy in XPA via PARP-1 hyperactivation and NAD^+^/SIRT1 reduction. Cell 157, 882–896. (10.1016/j.cell.2014.03.026) 24813611 PMC4625837

[47] Manandhar M, Lowery MG, Boulware KS, Lin KH, Lu Y, Wood RD 2017 Transcriptional consequences of XPA disruption in human cell lines. DNA Repair 57, 76–90. (10.1016/j.dnarep.2017.06.028) 28704716 PMC5731452

[48] Galluzzi L, Kepp O, Trojel-Hansen C, Kroemer G 2012 Mitochondrial control of cellular life, stress, and death. Circ. Res. 111, 1198–1207. (10.1161/CIRCRESAHA.112.268946) 23065343

[49] El-Khamisy SF 2011 To live or to die: a matter of processing damaged DNA termini in neurons. EMBO Mol. Med. 3, 78–88. (10.1002/emmm.201000114) 21246735 PMC3377058

[50] Rass U, Ahel I, West SC 2007 Defective DNA repair and neurodegenerative disease. Cell 130, 991–1004. (10.1016/j.cell.2007.08.043) 17889645

[51] Szymkowski DE, Yarema K, Essigmann JM, Lippard SJ, Wood RD 1992 An intrastrand d(GpG) platinum crosslink in duplex M13 DNA is refractory to repair by human cell extracts. Proc. Natl Acad. Sci. USA 89, 10 772–10 776. (10.1073/pnas.89.22.10772) PMC50424 1438274

[52] Kuraoka I, Bender C, Romieu A, Cadet J, Wood RD, Lindahl T 2000 Removal of oxygen free-radical-induced 5′,8-purine cyclodeoxynucleosides from DNA by the nucleotide excision-repair pathway in human cells. Proc. Natl Acad. Sci. USA 97, 3832–3837. (10.1073/pnas.070471597) 10759556 PMC18102

[53] Leon J, Sakumi K, Castillo E, Sheng Z, Oka S, Nakabeppu Y 2016 8-Oxoguanine accumulation in mitochondrial DNA causes mitochondrial dysfunction and impairs neuritogenesis in cultured adult mouse cortical neurons under oxidative conditions. Sci. Rep. 6, 22086 (10.1038/srep22086) 26912170 PMC4766534

[54] Clarke DD, Sokoloff L 1999 Circulation and energy metabolism of the brain. In Basic neurochemistry: molecular, cellular, and medical aspects, 6th edn (eds Siegel GJ, Agranoff BW, Albers RW, Fisher SK, Uhler MD), pp. 637–669 New York, NY: Lippincott-Raven.

[55] Krokan HE, Bjoras M 2013 Base excision repair. Cold Spring Harb Perspect. Biol. 5, a012583 (10.1101/cshperspect.a012583) 23545420 PMC3683898

[56] Reardon JT, Bessho T, Kung HC, Bolton PH, Sancar A 1997 *In vitro* repair of oxidative DNA damage by human nucleotide excision repair system: possible explanation for neurodegeneration in xeroderma pigmentosum patients. Proc. Natl Acad. Sci. USA 94, 9463–9468. (10.1073/pnas.94.17.9463) 9256505 PMC23224

[57] Brooks PJ et al. 2000 The oxidative DNA lesion 8,5'-(S)-cyclo-2'-deoxyadenosine is repaired by the nucleotide excision repair pathway and blocks gene expression in mammalian cells. J. Biol. Chem. 275, 22 355–22 362. (10.1074/jbc.M002259200) 10801836

[58] Sonohara Y, Yamamoto J, Tohashi K, Takatsuka R, Matsuda T, Iwai S, Kuraoka I 2019 Acetaldehyde forms covalent GG intrastrand crosslinks in DNA. Sci. Rep. 9, 660 (10.1038/s41598-018-37239-6) 30679737 PMC6345987

[59] Tudek B, Zdżalik-Bielecka D, Tudek A, Kosicki K, Fabisiewicz A, Speina E 2017 Lipid peroxidation in face of DNA damage, DNA repair and other cellular processes. Free Radical Biol. Med. 107, 77–89. (10.1016/j.freeradbiomed.2016.11.043) 27908783

[60] Wang AG, Xia T, Chu QL, Zhang M, Liu F, Chen XM, Yang KD 2004 Effects of fluoride on lipid peroxidation, DNA damage and apoptosis in human embryo hepatocytes. Biomed. Environ. Sci. 17, 217–222. 15386948

[61] Czerwińska J et al. 2018 ERCC1-deficient cells and mice are hypersensitive to lipid peroxidation. Free Radic. Biol. Med. 124, 79–96. (10.1016/j.freeradbiomed.2018.05.088) 29860127 PMC6098728

[62] Matsuda T, Kawanishi M, Yagi T, Matsui S, Takebe H 1998 Specific tandem GG to TT base substitutions induced by acetaldehyde are due to intra-strand crosslinks between adjacent guanine bases. Nucleic Acids Res. 26, 1769–1774. (10.1093/nar/26.7.1769) 9512551 PMC147446

[63] Alexandrov LB et al. 2013 Signatures of mutational processes in human cancer. Nature 500, 415–421. (10.1038/nature12477) 23945592 PMC3776390

[64] O'Shea KS, Kaufman MH 1979 The teratogenic effect of acetaldehyde: implications for the study of the fetal alcohol syndrome. J. Anat. 128, 65–76. 422485 PMC1232961

[65] Guerri C, Sanchis R 1985 Acetaldehyde and alcohol levels in pregnant rats and their fetuses. Alcohol 2, 267–270. (10.1016/0741-8329(85)90057-6) 4040377

[66] Levine EM 2004 Cell cycling through development. Development 131, 2241–2246. (10.1242/dev.01180) 15128665

[67] Nordman J, Orr-Weaver TL 2012 Regulation of DNA replication during development. Development 139, 455–464. (10.1242/dev.061838) 22223677 PMC3252349

[68] Lans H, Marteijn JA, Schumacher B, Hoeijmakers JHJ, Jansen G, Vermeulen W 2010 Involvement of global genome repair, transcription coupled repair, and chromatin remodeling in UV DNA damage response changes during development. PLoS Genet. 6, e1000941 (10.1371/journal.pgen.1000941) 20463888 PMC2865526

[69] Maslon MM et al. 2019 A slow transcription rate causes embryonic lethality and perturbs kinetic coupling of neuronal genes. EMBO J. 38, e101244 (10.15252/embj.2018101244) 30988016 PMC6484407

[70] Li W, Liu W, Kakoki A, Wang R, Adebali O, Jiang Y, Sancar A 2019 Nucleotide excision repair capacity increases during differentiation of human embryonic carcinoma cells into neurons and muscle cells. J. Biol. Chem. 294, 5914–5922. (10.1074/jbc.RA119.007861) 30808711 PMC6463700

[71] Friedberg EC, Meira LB 2006 Database of mouse strains carrying targeted mutations in genes affecting biological responses to DNA damage version 7. DNA Repair 5, 189–209. (10.1016/j.dnarep.2005.09.009) 16290067

[72] Snow ET 1997 The role of DNA repair in development. Reprod. Toxicol. 11, 353–365. (10.1016/S0890-6238(96)00148-7) 9100311

[73] Müller WA 1997 Model organisms in developmental biology. In Developmental biology (ed. Müller WA), pp. 21–121. New York, NY: Springer.

[74] Hubank M, Mayne L 1994 Expression of the excision repair gene, ERCC3 (excision repair cross-complementing), during mouse development. Dev. Brain Res. 81, 66–76. (10.1016/0165-3806(94)90069-8) 7805288

[75] Vinson RK, Hales BF 2001 Nucleotide excision repair gene expression in the rat conceptus during organogenesis. Mutat. Res. 486, 113–123. (10.1016/S0921-8777(01)00087-8) 11425516

[76] Notch EG, Mayer GD 2013 Impact of environmental estrogens on nucleotide excision repair gene expression in embryonic zebrafish. Comp. Biochem. Physiol. C Toxicol. Pharmacol. 157, 361–365. (10.1016/j.cbpc.2013.03.004) 23506788

[77] Menezo YJ, Russo G, Tosti E, El Mouatassim S, Benkhalifa M 2007 Expression profile of genes coding for DNA repair in human oocytes using pangenomic microarrays, with a special focus on ROS linked decays. J. Assist. Reprod. Genet. 24, 513–520. (10.1007/s10815-007-9167-0) 17899356 PMC3455023

[78] Lewandowski JP, Du F, Zhang S, Powell MB, Falkenstein KN, Ji H, Vokes SA 2015 Spatiotemporal regulation of GLI target genes in the mammalian limb bud. Dev. Biol. 406, 92–103. (10.1016/j.ydbio.2015.07.022) 26238476 PMC4587286

[79] Shimamoto T et al. 1995 Expression and functional analyses of the *Dxpa* gene, the *Drosophila* homolog of the human excision repair gene *XPA*. J. Biol. Chem. 270, 22 452–9. (10.1074/jbc.270.38.22452) 7673233

[80] Tomancak P et al. 2002 Systematic determination of patterns of gene expression during *Drosophila* embryogenesis. Genome Biol. 3, RESEARCH0088 (10.1186/gb-2002-3-12-research0088) 12537577 PMC151190

[81] Blackshaw S et al. 2004 Genomic analysis of mouse retinal development. PLoS Biol. 2, E247 (10.1371/journal.pbio.0020247) 15226823 PMC439783

[82] Reynaud E, Lomelí H, Vázquez M, Zurita M 1999 The *Drosophila melanogaster* homologue of the xeroderma pigmentosum D gene product is located in euchromatic regions and has a dynamic response to UV light-induced lesions in polytene chromosomes. Mol. Biol. Cell 10, 1191–1203. (10.1091/mbc.10.4.1191) 10198066 PMC25250

[83] Magdaleno S et al. 2006 BGEM: an in situ hybridization database of gene expression in the embryonic and adult mouse nervous system. PLoS Biol. 4, e86 (10.1371/journal.pbio.0040086) 16602821 PMC1413568

[84] Easterday MC et al. 2003 Neural progenitor genes. Germinal zone expression and analysis of genetic overlap in stem cell populations. Dev. Biol. 264, 309–322. (10.1016/j.ydbio.2003.09.003) 14651920

[85] Lipinszki Z, Kiss P, Pál M, Deák P, Szabó A, Hunyadi-Gulyas E, Klement E, Medzihradszky KF, Udvardy A 2009 Developmental-stage-specific regulation of the polyubiquitin receptors in *Drosophila melanogaster*. J. Cell Sci. 122, 3083–3092. (10.1242/jcs.049049) 19654212

[86] Diez-Roux G et al. 2011 A high-resolution anatomical atlas of the transcriptome in the mouse embryo. PLoS Biol. 9, e1000582 (10.1371/journal.pbio.1000582) 21267068 PMC3022534

[87] McWhir J, Selfridge J, Harrison DJ, Squires S, Melton DW 1993 Mice with DNA repair gene (ERCC-1) deficiency have elevated levels of p53, liver nuclear abnormalities and die before weaning. Nat. Genet. 5, 217–224. (10.1038/ng1193-217) 8275084

[88] de Vries A et al. 1995 Increased susceptibility to ultraviolet-B and carcinogens of mice lacking the DNA excision repair gene XPA. Nature 377, 169–173. (10.1038/377169a0) 7675086

[89] Mounkes LC, Jones RS, Liang BC, Gelbart W, Fuller MT 1992 A *Drosophila* model for xeroderma pigmentosum and Cockayne's syndrome: *haywire* encodes the fly homolog of *ERCC3*, a human excision repair gene. Cell 71, 925–937. (10.1016/0092-8674(92)90389-T) 1458540

[90] Andressoo J-O, Weeda G, de Wit J, Mitchell JR, Beems RB, van Steeg H, Van Der Horst GTJ, Hoeijmakers JH 2009 An Xpb mouse model for combined xeroderma pigmentosum and Cockayne syndrome reveals progeroid features upon further attenuation of DNA repair. Mol. Cell Biol. 29, 1276–1290. (10.1128/MCB.01229-08) 19114557 PMC2643825

[91] Sands AT, Abuin A, Sanchez A, Conti CJ, Bradley A 1995 High susceptibility to ultraviolet-induced carcinogenesis in mice lacking XPC. Nature 377, 162–165. (10.1038/377162a0) 7675084

[92] Cheo DL, Ruven HJ, Meira LB, Hammer RE, Burns DK, Tappe NJ, Van Zeeland AA, Mullenders LHF, Friedberg EC 1997 Characterization of defective nucleotide excision repair in XPC mutant mice. Mutat. Res. 374, 1–9. (10.1016/S0027-5107(97)00046-8) 9067411

[93] de Boer J, Donker I, de Wit J, Hoeijmakers JH, Weeda G 1998 Disruption of the mouse xeroderma pigmentosum group D DNA repair/basal transcription gene results in preimplantation lethality. Cancer Res. 58, 89–94. 9426063

[94] Li X, Urwyler O, Suter B 2010 Drosophila Xpd regulates Cdk7 localization, mitotic kinase activity, spindle dynamics, and chromosome segregation. PLoS Genet. 6, e1000876 (10.1371/journal.pgen.1000876) 20300654 PMC2837399

[95] Shiomi N, Mori M, Kito S, Harada YN, Tanaka K, Shiomi T 2005 Severe growth retardation and short life span of double-mutant mice lacking Xpa and exon 15 of Xpg. DNA Repair (Amst). 4, 351–357. (10.1016/j.dnarep.2004.10.009) 15661658

[96] Harada YN et al. 1999 Postnatal growth failure, short life span, and early onset of cellular senescence and subsequent immortalization in mice lacking the xeroderma pigmentosum group G gene. Mol. Cell. Biol. 19, 2366–2372. (10.1128/MCB.19.3.2366) 10022922 PMC84028

[97] Kamileri I, Karakasilioti I, Sideri A, Kosteas T, Tatarakis A, Talianidis I, Garinis GA 2012 Defective transcription initiation causes postnatal growth failure in a mouse model of nucleotide excision repair (NER) progeria. Proc. Natl Acad. Sci. USA 109, 2995–3000. (10.1073/pnas.1114941109) 22323595 PMC3286994

[98] Tian M, Shinkura R, Shinkura N, Alt FW 2004 Growth retardation, early death, and DNA repair defects in mice deficient for the nucleotide excision repair enzyme XPF. Mol. Cell. Biol. 24, 1200–1205. (10.1128/MCB.24.3.1200-1205.2004) 14729965 PMC321450

[99] Sekelsky JJ, McKim KS, Chin GM, Hawley RS 1995 The *Drosophila* meiotic recombination gene mei-9 encodes a homologue of the yeast excision repair protein Rad1. Genetics 141, 619–627. 8647398 10.1093/genetics/141.2.619 PMC1206761

[100] Ng JM et al. 2002 Developmental defects and male sterility in mice lacking the ubiquitin-like DNA repair gene mHR23B. Mol. Cell. Biol. 22, 1233–1245. (10.1128/MCB.22.4.1233-1245.2002) 11809813 PMC134644

[101] van der Horst GT et al. 2002 UVB radiation-induced cancer predisposition in Cockayne syndrome group A (*Csa*) mutant mice. DNA Repair (Amst). 1, 143–157. (10.1016/S1568-7864(01)00010-6) 12509261

[102] van der Horst GT et al. 1997 Defective transcription-coupled repair in Cockayne syndrome B mice is associated with skin cancer predisposition. Cell 89, 425–435. (10.1016/S0092-8674(00)80223-8) 9150142

[103] Sauerwald J, Soneson C, Robinson MD, Luschnig S 2017 Faithful mRNA splicing depends on the Prp19 complex subunit faint sausage and is required for tracheal branching morphogenesis in *Drosophila*. Development 144, 657–663. (10.1242/dev.144535) 28087625

[104] Yonemasu R, Minami M, Nakatsu Y, Takeuchi M, Kuraoka I, Matsuda Y, Higashi Y, Kondoh H, Tanaka K 2005 Disruption of mouse XAB2 gene involved in pre-mRNA splicing, transcription and transcription-coupled DNA repair results in preimplantation lethality. DNA Repair (Amst). 4, 479–491. (10.1016/j.dnarep.2004.12.004) 15725628

[105] Vinson RK, Hales BF 2002 DNA repair during organogenesis. Mutat. Res. 509, 79–91. (10.1016/S0027-5107(02)00223-3) 12427532

[106] Murai M, Enokido Y, Inamura N, Yoshino M, Nakatsu Y, van der Horst GT, Hoeijmakers JHJ, Tanaka K, Hatanaka H 2001 Early postnatal ataxia and abnormal cerebellar development in mice lacking xeroderma pigmentosum group A and Cockayne syndrome group B DNA repair genes. Proc. Natl Acad. Sci. USA 98, 13 379–13 384. (10.1073/pnas.231329598) PMC60879 11687625

[107] Merino C, Reynaud E, Vázquez M, Zurita M 2002 DNA repair and transcriptional effects of mutations in TFIIH in *Drosophila* development. Mol. Biol. Cell 13, 3246–3256. (10.1091/mbc.e02-02-0087) 12221129 PMC124156

[108] Stettler K, Li X, Sandrock B, Braga-Lagache S, Heller M, Dümbgen L, Suter B 2015 A *Drosophila* XPD model links cell cycle coordination with neuro-development and suggests links to cancer. Dis. Models Mech. 8, 81–91. 10.1242/dmm.016907 PMC4283652 25431422

[109] Silva IA, Cancela ML, Conceicao N 2012 Molecular cloning and expression analysis of *xpd* from zebrafish (*Danio rerio*). Mol. Biol. Rep. 39, 5339–5348. (10.1007/s11033-011-1333-x) 22187342

[110] Lee TI, Young RA 2013 Transcriptional regulation and its misregulation in disease. Cell 152, 1237–1251. (10.1016/j.cell.2013.02.014) 23498934 PMC3640494

[111] Vermeulen W, Jaeken J, Jaspers NG, Bootsma D, Hoeijmakers JH 1993 Xeroderma pigmentosum complementation group G associated with Cockayne syndrome. Am. J. Hum. Genet. 53, 185–192. 8317483 PMC1682247

[112] Sekelsky JJ, Hollis KJ, Eimerl AI, Burtis KC, Hawley RS 2000 Nucleotide excision repair endonuclease genes in *Drosophila melanogaster*. Mutat. Res. 459, 219–228. (10.1016/S0921-8777(99)00075-0) 10812334

[113] Calléja FM, Nivard MJ, Eeken JC 2001 Induced mutagenic effects in the nucleotide excision repair deficient *Drosophila* mutant *mus201^D1^*, expressing a truncated XPG protein. Mutat. Res. 461, 279–288. (10.1016/S0921-8777(00)00055-0) 11104904

[114] Trego KS et al. 2016 Non-catalytic roles for XPG with BRCA1 and BRCA2 in homologous recombination and genome stability. Mol. Cell 61, 535–546. (10.1016/j.molcel.2015.12.026) 26833090 PMC4761302

[115] Vermeij WP et al. 2016 Restricted diet delays accelerated ageing and genomic stress in DNA-repair-deficient mice. Nature 537, 427–431. (10.1038/nature19329) 27556946 PMC5161687

[116] Jaarsma D, van der Pluijm I, van der Horst GTJ, Hoeijmakers JHJ 2013 Cockayne syndrome pathogenesis: lessons from mouse models. Mech. Ageing Dev. 134, 180–195. (10.1016/j.mad.2013.04.003) 23591128

[117] Sacco R, Tamblyn L, Rajakulendran N, Bralha FN, Tropepe V, Laposa RR 2013 Cockayne syndrome b maintains neural precursor function. DNA Repair 12, 110–120. (10.1016/j.dnarep.2012.11.004) 23245699

[118] Karikkineth AC, Scheibye-Knudsen M, Fivenson E, Croteau DL, Bohr VA 2017 Cockayne syndrome: clinical features, model systems and pathways. Ageing Res. Rev. 33, 3–17. (10.1016/j.arr.2016.08.002) 27507608 PMC5195851

[119] Ciaffardini F, Nicolai S, Caputo M, Canu G, Paccosi E, Costantino M, Frontini M, Balajee AS, Proietti-De-Santis L 2014 The Cockayne syndrome B protein is essential for neuronal differentiation and neuritogenesis. Cell Death Dis. 5, e1268 (10.1038/cddis.2014.228) 24874740 PMC4047889

[120] Lee MH, Ahn B, Choi IS, Koo H-S 2002 The gene expression and deficiency phenotypes of Cockayne syndrome B protein in *Caenorhabditis elegans*. FEBS Lett. 522, 47–51. (10.1016/S0014-5793(02)02880-6) 12095617

[121] Babu V, Hofmann K, Schumacher B 2014 A *C. elegans* homolog of the Cockayne syndrome complementation group A gene. DNA Repair 24, 57–62. (10.1016/j.dnarep.2014.09.011) 25453470 PMC4255241

[122] Lans H, Vermeulen W 2011 Nucleotide excision repair in *Caenorhabditis elegans*. Mol. Biol. Int. 2011, 542795 (10.4061/2011/542795) 22091407 PMC3195855

[123] Kersey PJ et al. 2018 Ensembl genomes 2018: an integrated omics infrastructure for non-vertebrate species. Nucleic Acids Res. 46, D802–D808. (10.1093/nar/gkx1011) 29092050 PMC5753204

[124] de Cock JG, Klink EC, Lohman PH, Eeken JC 1992 Absence of strand-specific repair of cyclobutane pyrimidine dimers in active genes in *Drosophila melanogaster* Kc cells. Mutat. Res. 274, 85–92. (10.1016/0921-8777(92)90055-8) 1378210

[125] van der Helm PJ, Klink EC, Lohman PH, Eeken JC 1997 The repair of UV-induced cyclobutane pyrimidine dimers in the individual genes Gart, Notch and white from isolated brain tissue of *Drosophila melanogaster*. Mutat. Res. 383, 113–124. (10.1016/S0921-8777(96)00050-X) 9088344

[126] Sekelsky J 2017 DNA repair in *Drosophila*: mutagens, models, and missing genes. Genetics 205, 471–490. (10.1534/genetics.116.186759) 28154196 PMC5289830

[127] Teng Y, Waters R 2000 Excision repair at the level of the nucleotide in the upstream control region, the coding sequence and in the region where transcription terminates of the *Saccharomyces cerevisiae* MFA2 gene and the role of RAD26. Nucleic Acids Res. 28, 1114–1119. (10.1093/nar/28.5.1114) 10666451 PMC102608

[128] Li S, Smerdon MJ 2002 Rpb4 and Rpb9 mediate subpathways of transcription-coupled DNA repair in *Saccharomyces cerevisiae*. EMBO J. 21, 5921–5929. (10.1093/emboj/cdf589) 12411509 PMC131086

[129] Evans JM, Day JP, Cabrero P, Dow JA, Davies SA 2008 A new role for a classical gene: white transports cyclic GMP. J. Exp. Biol. 211, 890–899. (10.1242/jeb.014837) 18310115

[130] Ferreiro MJ, Perez C, Marchesano M, Ruiz S, Caputi A, Aguilera P, Barrio R, Cantera R 2017 *Drosophila melanogaster white* mutant *w^1118^* undergo retinal degeneration. Front. Neurosci. 11, 732 (10.3389/fnins.2017.00732) 29354028 PMC5758589

[131] Wilson DM, Rieckher M, Williams AB, Schumacher B 2017 Systematic analysis of DNA crosslink repair pathways during development and aging in *Caenorhabditis elegans*. Nucleic Acids Res. 45, 9467–9480. (10.1093/nar/gkx660) 28934497 PMC5766164

